# Variation in genes encoding eosinophil granule proteins in atopic dermatitis patients from Germany

**DOI:** 10.1186/1477-5751-7-9

**Published:** 2008-11-13

**Authors:** Qumar Parwez, Susanne Stemmler, Jörg T Epplen, Sabine Hoffjan

**Affiliations:** 1 Private medical practice, Gladbeck, Germany; 2 Department of Human Genetics, Ruhr-University, Bochum, Germany

## Abstract

**Background:**

Atopic dermatitis (AD) is believed to result from complex interactions between genetic and environmental factors. A main feature of AD as well as other allergic disorders is serum and tissue eosinophilia. Human eosinophils contain high amounts of cationic granule proteins, including eosinophil cationic protein (ECP), eosinophil-derived neurotoxin (EDN), eosinophil peroxidase (EPO) and major basic protein (MBP). Recently, variation in genes encoding eosinophil granule proteins has been suggested to play a role in the pathogenesis of allergic disorders. We therefore genotyped selected single nucleotide polymorphisms within the *ECP, EDN, EPO *and *MBP *genes in a cohort of 361 German AD patients and 325 healthy controls.

**Results:**

Genotype and allele frequencies did not differ between patients and controls for all polymorphisms investigated in this study. Haplotype analysis did not reveal any additional information.

**Conclusion:**

We did not find evidence to support an influence of variation in genes encoding eosinophil granule proteins for AD pathogenesis in this German cohort.

## Background

Atopic dermatitis (AD) is a chronic inflammatory skin disease believed to arise from complex interactions between genetic and environmental factors [1]. AD belongs to the group of allergic disorders, also including allergic asthma, allergic rhinitis and food allergy. As a main feature of allergic disorders, AD is often accompanied by eosinophilia [2]. Human eosinophils contain high amounts of cationic granule proteins, including eosinophil cationic protein (ECP), eosinophil-derived neurotoxin (EDN), eosinophil peroxidase (EPO) and major basic protein (MBP). Eosinophil degranulation with deposition of these proteins in the skin has been shown to play an important role in AD [2]. Furthermore, serum and urine concentrations of eosinophil proteins are indirect measures of inflammatory activity in AD. For example, several studies have confirmed that urine EDN (also called EPX) is a useful *in vitro *parameter of inflammation in AD [3-5]. Measurement of ECP levels in serum also is a frequently used tool in monitoring AD activity [4,6].

Recently, variation in genes encoding eosinophil granule proteins has been suggested as potential factor in the pathogenesis of allergic disorders. In particular, a non-synonymous polymorphism in the *ECP *gene was associated with allergic symptoms [7], and a polymorphism in the 3'UTR of this gene showed correlation with the cellular content of ECP [8]. More recently, *ECP *haplotypes were found associated with asthma and serum ECP levels [9]. Variation in the *EDN *gene was evaluated for an association with tropical pulmonary eosinophilia but did not show an association with this disease in a small cohort [10].

Since eosinophil degranulation seems to play an important role in AD, we speculated that variation in genes encoding eosinophil granule proteins might show an association with AD. We therefore genotyped selected single nucleotide polymorphisms (SNPs) within the *ECP, EDN, EPO *and *MBP *genes in a cohort of 361 German AD patients and 325 healthy controls.

## Methods

### Subjects

361 unrelated patients with AD, including 217 children and 144 adults, were recruited by a consultant specialist for AD (Q.P., Gladbeck, Germany). Mean age of the AD patients was 18 ± 7 years. The AD diagnosis was based on the criteria developed by Hanifin and Rajka [11]. 325 control samples from adults of at least 40 years of age without known allergies, asthma or AD were collected in the same private practice as the AD patients. Mean age of the controls was 57 ± 13 years. We specifically chose to use non-allergic adults as controls because the risk remains very high for primarily asymptomatic children to develop an allergic disease during childhood or even adulthood [12,13]. The control subjects underwent clinical examination in order to exclude symptoms of AD, asthma or allergic rhinitis. They further reported to have no allergic symptoms and no first degree relatives with known allergic diseases. All patients and control subjects were Germans of European origin. Informed consent was obtained from all subjects. The study was approved by the Ethics Committee of the University of Bochum, and the Declaration of Helsinki protocols were followed.

### Genotyping

Genotyping for all polymorphisms was performed by polymerase chain reaction (PCR) with subsequent restriction enzyme digestion. Details for PCR conditions and restriction enzymes are summarized in table 1. PCR reactions were performed in a total volume of 10 μl, containing 45 ng DNA, 2 mM of each dNTP, 1–3 mmol MgCl_2_, 5 pmol of the respective forward and reverse primer and 0.4 U Taq polymerase (Ares Bioscience, Lüdinghausen, Germany) on a Biometra Thermal Cycler (Göttingen, Germany). The PCR products were digested with the respective enzyme (table 1, 0.01 U/ng DNA) at 37°C for three hours. The fragments were subsequently separated on 2.5–3.5% agarose gels in 1×TBE buffer (30 min, 200 V) and visualized using ethidium bromide staining.

**Table 1:** Polymorphisms typed in the *EDN, ECP, EPO *and *MBP *genes.

**Gene**	**Rs number**	**Location**	**Amino acid**	**Primer**	**Annealing temperature**	**Restriction enzyme**
*EDN*	rs2013109	Intron 1	-	F: GGGTAAGTCAACGATCCCCAG R: GGTCTTGGTTATGACACACACTGTAGT	69°C	HpyF10VI
*EDN*	rs10132319	Intron 1	-	F: GGGTAAGTCAACGATCCCCAG R: GGTCTTGGTTATGACACACACTGTAGT	65°C	AciI
*ECP*	rs17792481	Intron 1	-	F: TGAGGGAGAGGTGAGCTGAAGT R: TGATGTGCTGGATGGCAAAC	58°C	MboI
*ECP*	rs2073342	Exon 2	T124R	F: TACGCTGCCCTCATAACAGAACT R: GAACTGGAACCACAGGATACCG	58°C	PstI
*ECP*	rs2233860	3' UTR	-	F: GTATGCAGACAGACCAGGAAGGA R: TTGGCAGATGAGTGATGATGAGTA	61°C	RsaI
*EPO*	rs11652709	Exon 4	Q122H	F: CTACCCTGGCCTGGAGTAGAAG R: CACGCACTTGTTGTTGCACC	64°C	HpyF10VI
*EPO*	rs3785496	Intron 6	-	F: ACTGGTTGTCACTTCCCCTCTC R: CAAAACCTTGCTTAAAACTCCCTT	58°C	MseI
*MBP*	rs490358	Exon 2	-	F: GGAAAAAACAGAGAAAGAAAGACTGAA R: CACTCCCACGGTCCCCTAAT	58°C	BslI
*MBP*	rs3741098	Intron 3	-	F: TGGTGGACAAAAACCTTACGTGTC R: ATGCTGGGGGCATATCCGA	58°C	BsaHI

### Statistical analyses

Genotype and allele frequencies were ascertained by direct counting and subsequently analyzed according to the χ^2 ^method. Deviations from Hardy-Weinberg equilibrium were evaluated using the DeFinetti program [14]. P < 0.05 was considered to be significant. Haplotype frequencies were estimated using the Haploview software and compared between cases and controls using a contingency χ^2 ^test. We performed power analyses with the Genetic Power Calculator program [15].

## Results

We genotyped a total of nine SNPs in four genes encoding eosinophil granule proteins (table 1). rs10132319 in intron 1 of the *EDN *gene turned out to be monomorphic in our cohort and thus was excluded from further analysis. All other SNPs were in Hardy-Weinberg equilibrium in cases and controls. None of the polymorphisms investigated here showed an association with AD in our cohort (table 2). Power analyses performed with the Genetic Power Calculator program [15] revealed, that given a multiplicative model with a genotypic relative risk of 2 for heterozygotes and 4 for homozygotes and a D' of 0.9, we have 89% power to detect a potential effect. Choosing more conservative parameters with a genotypic relative risk of 1.5/2.25 and D' of 0.8, would yield only 33% power.

**Table 2:** Genotype frequencies of *EDN, ECP, EPO *and *MBP *polymorphisms in AD patients and controls.

**Gene**	**Polymorphism**	**Genotypes**	**AD patients**	**controls**	**p-value**
*EDN*	rs2013109	C/C	22 (6.1%)	23 (7.1%)	0.66
		C/G	148 (41.0%)	123 (37.8%)	
		G/G	191 (52.9%)	179 (55.1%)	
					
	rs10132319	Not polymorphic	-	-	-
					
*ECP*	rs2073342	A/A	53 (14.7%)	57 (17.6%)	0.45
		A/C	185 (51.2%)	152 (47.1%)	
		C/C	123 (34.1%)	114 (35.3%)	
					
	rs2073342	C/C	26 (7.2%)	32 (9.8%)	0.13
		C/G	158 (43.9%)	120 (36.9%)	
		G/G	176 (48.9%)	173 (53.2%)	
					
	rs2233860	C/C	15 (4.1%)	19 (5.9%)	0.55
		C/G	123 (34.1%)	104 (32.1%)	
		G/G	223 (61.8%)	201 (62.0%)	
					
*EPO*	rs11652709	C/C	34 (9.4%)	35 (10.8%)	0.42
		C/G	170 (47.2%)	137 (42.3%)	
		G/G	156 (43.3%)	152 (46.9%)	
					
	rs3785496	G/G	23 (6.4%)	18 (5.6%)	0.89
		G/A	142 (39.4%)	130 (40.1%)	
		A/A	195 (54.2%)	176 (54.3%)	
					
*MBP*	rs490358	A/A	29 (8.0%)	27 (8.3%)	0.98
		A/G	137 (38.0%)	121 (37.2%)	
		G/G	195 (54.0%)	177 (54.5%)	
					
	rs3741089	T/T	99 (27.6%)	99 (30.7%)	0.66
		T/C	184 (51.2%)	158 (49.1%)	
		C/C	76 (21.2%)	65 (20.2%)	

Haplotype analyses in the *ECP *gene revealed four different haplotypes with a frequency above 1%: A-G-G, C-G-G, C-C-C and C-C-G, as described before [9]. Haplotype frequencies did not differ between AD patients and controls (table 3).

**Table 3:** Frequencies and p-values of *ECP *haplotypes in AD patients and controls.

**Haplotype**	**Frequency in AD patients (n = 361)**	**Frequency in controls (n = 325)**	**p-value**
A-G-G	0.401	0.408	0.762
C-G-G	0.305	0.304	0.934
C-C-C	0.209	0.214	0.830
C-C-G	0.080	0.070	0.439

## Discussion

In the present study, we did not find evidence to support an influence of variation in genes encoding eosinophil granule proteins for AD pathogenesis in a thoroughly characterized German cohort.

In the *ECP *gene, the three polymorphisms were evaluated that had shown association to allergic phenotypes either in single SNP [7] or haplotype analysis [9] in previous studies. Genotype as well as haplotype frequencies in our cohort were very similar to the frequencies reported in these other studies, but did not differ between AD patients and controls. Thus, our results seem to contradict these earlier reports. Yet, none of the preceding studies specifically addressed the phenotype AD, but rather allergic symptoms in general [7] or allergic asthma [9]. Linkage studies have shown that there is surprisingly little overlap between the main susceptibility regions for asthma and AD, even though both diseases belong to the group of allergic disorders [16]. Thus, it is possible that *ECP *variation might influence the development of (allergic) asthma but have no measurable influence on AD development. Furthermore, the association of the *ECP *T124R SNP with allergic symptoms was demonstrated in a very small cohort (209 medical students and 76 asthmatic patients) [7], and it still remains to be investigated whether this association will hold true in a larger cohort of allergic patients. Clearly, additional studies in sufficiently large cohorts of patients with allergy, asthma and AD are needed to further elucidate the role of variation in this gene for the various allergic diseases.

For the other three genes evaluated in this study, no association studies for AD or asthma have been published so far. Only the *EDN *gene (together with *ECP*) was evaluated for an association with tropical pulmonary eosinophilia but did not show an association with this disease in a small cohort [10]. We nevertheless speculated that they might constitute interesting candidate genes for AD since eosinophil degranulation with deposition of all of these proteins in the skin has been shown to play an important role in AD [2]. Furthermore, several studies have confirmed that urine EDN is a useful *in vitro *parameter of inflammation in AD [3-5]. Interestingly, the *EPO *gene is located on chromosome 17q23, close to the regions of highest linkage to AD or AD severity in two genome-wide screens [17,18]. Yet, none of the investigated polymorphisms in these genes showed differences in allele, genotype or haplotype distribution between AD patients and controls in our cohorts, suggesting that they may probably not play an important role in AD pathogenesis.

## Conclusion

We did not find evidence to support an influence of variation in genes encoding eosinophil granule proteins for AD pathogenesis in this German cohort. However, we cannot exclude a contribution of variation in these genes entirely. Additional association studies as well as functional assessment of the investigated polymorphisms might further elucidate their role in allergic diseases.

## Abbreviations

AD: atopic dermatitis; ECP: eosinophil cationic protein; EDN: eosinophil derived neurotoxin; EPO: eosinophil peroxidase; MBP: major basic protein; PCR: polymerase chain reaction

## Competing interests

The authors declare that they have no competing interests.

## Authors' contributions

QP recruited the patients and control subjects, collected clinical data, performed the experiments and drafted the manuscript. SS and JTE participated in the design and coordination of the study. SH was in charge of the design and coordination of the study as well as the statistical analyses and finalised the manuscript. All authors read and approved the final manuscript.
